# Regional variation in overweight and associations with regional profiles using a Japanese national open-source database

**DOI:** 10.1371/journal.pone.0328435

**Published:** 2025-08-25

**Authors:** Megumi Yoshigai, Etsu Goto, Daisuke Takada, Yuichi Imanaka

**Affiliations:** 1 Department of Healthcare Economics and Quality Management, School of Public Health, Kyoto University Graduate School of Medicine, Kyoto, Japan; 2 Bordeaux Population Health, U1219, University of Bordeaux, Bordeaux, France; 3 Department of Food Science and Nutrition, Faculty of Human Life and Science, Doshisha Woman’s College of Liberal Arts, Kyoto, Japan; 4 Department of Health Security System, Centre for Health Security, Kyoto University Graduate School of Medicine, Kyoto, Japan; Niigata University, JAPAN

## Abstract

The objectives of this study were to describe regional variation in overweight and to investigate factors associated with overweight at the secondary medical area (SMA) level, accounting for regional economic sector profiles. We utilized data from the specific health checkup, which targets individuals aged 40–74 years. Following descriptive analyses, we employed partial least squares regression analyses using an open-access version of specific health checkup data from the National Database of Health Insurance Claims and Specific Health Checkups of Japan. This approach allowed us to identify latent variables related to regional variation in overweight and to examine associated lifestyle and socioenvironmental factors. Identifying these latent variables helps uncover underlying regional or socioeconomic patterns not directly observable, thereby informing more targeted public health interventions. The distinct characteristics of areas associated with a higher proportion of overweight persons were identified—the key latent variable encompassing low socioeconomic status and a high proportion of family workers. Additionally, our findings suggested that the drinking and eating environment may present challenges in regions with a high proportion of workers in the information and real estate sectors. In contrast, reduced walkability of the environment may be problematic in regions with many workers in the manufacturing and transportation sectors. Our study underscores the importance of addressing the unique challenges faced by each area with attention given to their local traits and industrial structures, which may have unconsciously shaped residents’ lifestyles and daily behaviors. As area-level variation in overweight and obesity related to contextual factors, such as economic sector profiles, have not been extensively studied internationally, this study provides a valuable insight into research on factors associated with overweight.

## Introduction

The value of monitoring trends and inequalities in overweight and obesity and developing preventive interventions have been recognized as global priorities [1,2] due to the financial burden that they impose [3,4] and the health consequences that ensue, even in the absence of metabolic risk factors [5]. As of 2022, 2.5 billion persons aged 18 years and over were overweight, a condition characterized by excessive fat deposits and a body mass index (BMI) of 25 kg/m^2^ or greater, of which 890 million were classified as obese, defined by a BMI of 30 kg/m^2^ or greater [1]. The health consequences of overweight and obesity include cardiovascular diseases, diabetes, neurological disorders, cancers, chronic respiratory diseases, and digestive disorders [1], which are often referred to as lifestyle diseases as their risk factors are closely related to a person’s lifestyle. A report from Japan showed that, in 2019, the proportion of men aged 20 years and over who were overweight ranged between 23% in 20s and 40% in 40s, nearly double the proportion recorded in 1975, while the proportion of women with overweight remained almost unchanged [6]. Alongside these worldwide trends, substantial emphasis has been directed toward improving individual behaviors, such as increased physical activity, improved sleep, and better dietary habits [1,7]. However, given the spatial clustering of obesity, previous studies also highlight the importance of environmental risk factors [1,2]. Environmental factors associated with health are not limited to biological, physical, and chemical exposures; social exposures at the workplace or community level also play crucial roles [8]. Increasing attention has also been given to the roles that corporations and governments play in promoting healthy lifestyles [9], owing to the challenges of incorporating exercise and healthy eating habits amidst work constraints [10] and environmental factors [11].

The benefits of a population-level approach, promoting unconscious behavioral changes, have been demonstrated, considering the contextual characteristics of specific geographic areas [12–14]. However, area-level variation in overweight and obesity, especially those linked to contextual factors such as economic sector profiles [15,16], have not been extensively studied internationally. As an initial step, this study focused on Japan. The first objective of this study was to describe the distribution of overweight persons based on the annual specific health checkup data categorized by secondary medical areas (SMAs). The second objective was to identify the latent variables (LVs) relating to regional variation in overweight and examine the associated factors accounting for lifestyle and socioenvironmental structures, including regional economic sector profiles. By enhancing our understanding of regional variation in overweight and their associated characteristics, our study will inform the development of future interventions that promote healthy lifestyles, manage body weight, and prevent lifestyle-related diseases.

## Materials and methods

### Data sources

We utilized an open-access version of specific health checkup data from the National Database of Health Insurance Claims and Specific Health Checkups of Japan (NDB). This health checkup targets individuals aged 40–74 years. We accessed the data on 31^st^, October, 2023, with no information that could identify individual participants. The NDB is a national administrative claims database established by the Ministry of Health, Labour, and Welfare of Japan primarily to support the regulation of national health expenditure and inform health policy planning [17]. We incorporated data from other public sources to obtain regional socioenvironmental variables (Table 1) via the portal site for Japanese Government Statistics. To improve readability, variables are grouped into conceptual categories in Table 1; however, each variable was analyzed separately in the models. Additional details, including definitions and data sources, are provided in S1 Table in S1 File.

**Table 1:** Summary of lifestyle and socioenvironmental variables and data sources.

Lifestyle variables	Data source
Proportion of people with specific lifestyle behavior (%)	Open-access version of specific health checkups data (2018)
**Socioenvironmental variables**	**Data source**
Proportion of workers in each industrial classification (%)	Population census of Japan (2015)
Proportion of each employment status (%)	
Proportion of workers inside or outside their local city or prefecture (%)	
Proportion of each commuting method (%)	Population census of Japan (2010)
Proportion of each educational level (%)	
Taxable income per taxpayer (1,000 yen)	Municipal tax survey^*^ (2018)
Tax revenue from tabacco tax per people aged 20 and older (1,000 yen)	Municipal tax collection survey^*^ (2015)
	Population census of Japan (2010)
Proportion of each household type (%)	Population census of Japan (2015)
Population Aged 60 and Above (%)	Basic Resident Registration (2018)
Access to each shop per km^2^	Economic census (2016)
	Population census of Japan, Municipalities Area Statistics of Japan (2015)
Access to each health service per 100,000 people	Survey of medical institutions, hospital reports (2015)
	Population census of Japan (2015)
	Survey of institutions and establishments for long-term care (2015)
	Population census of Japan (2015)
Population density (people per km^2^)	Population census of Japan (2015)
	Population census of Japan, Municipalities Area Statistics of Japan (2015)
Proportion of areas for city planning (%)	Urban planning annual report^*^
	Population census of Japan, Municipalities Area Statistics of Japan (2015)

* The survey name was translated to English by authors.

Specific health checkups in Japan are a legally mandated annual screening for individuals aged 40–74 years, aimed at preventing the onset or worsening of lifestyle-related diseases [18]. Based on health checkup results, health insurance societies provide specific health guidance programs to individuals, namely, intensive health guidance or less intensive motivation-enhancing guidance [19]. The specific health guidance program is determined based on the person’s waist circumferences, BMI, blood sugar, cholesterol, blood pressure, and smoking habits [20]. Although we use the term “overweight” following the World Health Organization’s definition (BMI ≥ 25 kg/m^2^) for clarity and consistency with international standards, we should note that a BMI of 25 kg/m^2^ or greater is classified as obesity in Japan [6].

### Units of analysis

The units of our analysis were SMAs, which are defined by Japan’s Medical Care Act. The SMAs are 335 regions at the sub-prefecture level, each comprising several municipalities [21]. These regions are organized to provide comprehensive inpatient, outpatient, and long-term care, considering geographical factors and the residents’ access to daily necessities [22]. Data originally organized at the municipality level were reorganized into SMAs as required. We also used 11 regional classifications defined by the Japanese Cabinet Office [23] for visualization purposes in some supplementary figures.

### Statistical analysis

First, we described the distribution of overweight in Japan by SMAs, which aligns with our first objective. Second, we used a partial least squares (PLS) regression analysis with a proportion of overweight individuals in each SMA as objective variables to address our second objective. Originally developed in the social sciences and later widely adopted in chemometrics, PLS regression is widely recognized for its usefulness in predicting a set of dependent variables from a large set of independent variables. This model identifies latent components that exhibit the strongest covariance with the outcome variable and utilizes them in regression analysis [24]. All analyses were conducted separately for men and women, considering the potential differences in factors associated with overweight and LVs by sex based on the sex categories assigned in the original data (Table 1), when available. We believed the separate analyses could offer meaningful insights for developing future hypotheses and interventions. We employed BMI as the outcome, being the most commonly adopted measure [11]. Persons with a BMI of 25 kg/m^2^ or higher were identified from the specific health checkup data in 2018. The proportion of overweight in the population was calculated at the SMA level and directly standardized at five-year intervals based on the model population from a basic resident registration in 2018. The selection of explanatory variables was based on conceptual frameworks from *Obesity Mapping: A Conceptual Framework Deliverable 4.4* [8]. Focusing on the individual, work/school/home, and area-level factors in the conceptual model, we included mainly two types of factor in our explanatory variables: lifestyle and socioenvironmental factors (Table 1). For lifestyle, we used the area-level proportions of individuals with specific lifestyle characteristics. Although there was limited data available for socioenvironmental factors, we included factors related to work, living environment, family, access to health services, and access to food. As work-related factors, we included mean taxable income per taxpayer, the proportion of unemployed working-age individuals in the population, and workers in each industrial classification [25] that may reflect the regional economic sector profile and commuters using specific modes of transportation in each SMA. Factors related to the living environment consisted of population density, urbanization promotion areas, urbanization control areas, commercial, quasi-industrial, industrial, or exclusively industrial areas per square kilometer of habitable living area, and mean tobacco tax revenue per taxpayer aged 20 years and over. The proportion of each household type was used to represent family factors, while the number of clinics and shops per square kilometer of habitable living area were used to indicate access to health services and food.

We used a leave-one-out cross-validation approach to evaluate the model by identifying the number of factors required to minimize the root mean squared error of prediction (RMSEP) [26]. Using the pls package in R, we selected the number based on a one-sigma strategy [27] and then plotted each LV’s x-score and objective variables. Cross-validation using the one-sigma heuristic suggested five components for men’s and eight components for women’s model (S1 and S2 Figs). The cumulative explained variances are reported in S3 and S4 Figs. For interpretation in the result section, we selected three latent variables. We would like to emphasize that our objective was not to create prediction models but to identify LVs associated with regional overweight variation and describe LVs and their loading factors. We have listed only three LVs in the Results section due to space constraints and interpretability considerations based on the plots and improvements in the cumulative explained variance of the dependent variables by each LV. We also visualized the scores of LV1 and LV2 with the proportion of overweight individuals in each SMA, which was colored based on the 11 regional classification defined by the Japanese Cabinet Office [23].

### Ethical considerations

The Ethics Committee of Kyoto University Graduate School and Faculty of Medicine approved this study (R2215-2).

## Results

### Regional distribution of overweight

**Fig 1:**
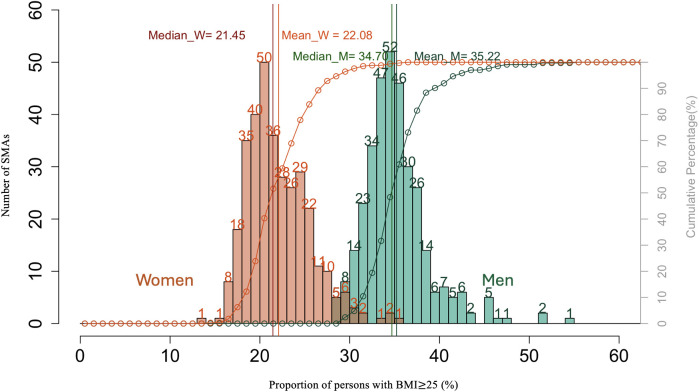
Histograms of the distribution of SMAs by the proportion of men and women aged 40-74 with overweight (BMI ≥ 25 kg/m^2^).

Descriptive statistics for SMA-level lifestyle and socioenvironmental explanatory variables are summarized in S2 and S3 Tables in S1 File. Fig 1 shows the distribution of overweight across the 335 SMAs, presented as a frequency histogram illustrating how many regions fall into each overweight proportion category. The mean proportions of overweight in men and women aged 40–74 years for all SMAs were 35% ± 3.6% and 22% ± 3.5%, respectively. The proportions ranged between 29%–55% for men and 14%–36% for women. The SMAs in Okinawa had the three highest values for both men and women, while the lowest proportions were observed in SMAs in Tokyo for women and in Gifu for men.

The mean proportions of overweight in men and women aged 40–74 years were 35% ± 3.6% and 22% ± 3.5%. Okinawa had the three highest values for both men and women, while the lowest proportions were observed in SMAs in Tokyo for women and in Gifu for men.

### PLS regression analysis

There were five extracted LVs for men and eight for women. Factor loadings are listed in Tables 2 and 3. In summary, the identified LVs captured key characteristics of areas, which were interpreted as lower education and income levels and a higher proportion of family workers, a higher proportion of the population living alone combined with unfavorable eating and drinking habits, and a less walkable environment. We will first investigate the details of LVs in men. The LV1 was positively loaded by socioenvironmental factors such as the proportion of persons with a lower level of educational attainment (junior high school or high school) and residents working in their home municipality, agroforestry, compound services, and construction sectors, and by lifestyle factors such as smokers, and those with difficulty chewing food (Table 2). The main negatively loaded factors at the SMA level were taxable income and workers in the information and communications sector. Negative loadings indicate that these variables are inversely associated with the LV1. The main factors contributing to LV2 included both lifestyle and socioenvironmental factors, such as skipping breakfast, drinking, over 10 kg in body weight gain relative to their body weight in their 20s, the regional proportion of single-person households, non-relative households, workers in the real estate or information and communications sectors, and the number of shops or restaurants. Population density had relatively higher loading than other variables. At an SMA level, the LV3 was negatively loaded by the proportion of the number of general and dental clinics, single-person households, workers in medical, accommodation (hospitality), or food service sectors, board members, and commercial areas, while positively loaded by the proportion of workers in the manufacturing industry, urban control areas and exclusively industrial areas.

**Table 2:** Factor loadings for men’s model.

	LV1	LV2	LV3
Proportion of persons with smoking habit	0.186	−0.028	0.096
Proportion of persons with no physical activity for more than one hour a day	−0.044	−0.085	0.066
Proportion of persons with no exercise for more than two weeks	0.060	−0.121	0.024
Proportion of persons who have dinner within two hours before bedtime more than 3 times a week	−0.173	0.139	0.087
Proportion of persons who drink alcohol everyday	0.098	−0.140	−0.151
Proportion of persons who drink more than 3 glasses (540 ml) of alcohol per day	0.082	0.168	−0.037
Proportion of persons who drink about 2–3 glasses (360–540 ml) of alcohol per day	0.119	0.102	−0.042
Proportion of persons who drink about 1–2 glasses (180–360 ml) of alcohol per day	0.115	−0.087	−0.028
Proportion of persons who drink less than 1 glass (180 ml) of alcohol per day	−0.155	−0.064	0.051
Proportion of persons who snack or drink sweet beverages between meals everyday	−0.029	0.028	0.018
Proportion of persons who sometimes snack or drink sweet beverages between meals	0.035	0.005	0.179
Proportion of persons who skip breakfast more than 3 times a week	0.031	0.234	−0.008
Proportion of persons who are unable to sleep well and enough	−0.202	0.044	0.080
Proportion of persons who had over 10 kg in body weight gain relative to their body weight in their 20s	0.032	0.198	0.191
Proportion of persons who walk at a faster pace than others of the same age	−0.110	0.143	−0.084
Proportion of persons who are unable to chew food due to problems of tooth, gum, or occlusion	0.018	0.019	0.115
Proportion of persons with with difficulty chewing food due to problems of tooth, gum, or occlusion	0.186	−0.073	0.024
Proportion of persons who eat quicker than others	−0.071	0.107	−0.127
Proportion of persons who eat slower than others	−0.062	−0.038	−0.021
Proportion of workers in agriculture and forestry	0.205	−0.099	−0.056
Proportion of workers in fisheries	0.176	0.022	−0.037
Proportion of workers in mining and quarrying	0.149	−0.029	−0.011
Proportion of workers in construction	0.217	−0.045	0.027
Proportion of workers in manufacturing	−0.185	−0.106	0.203
Proportion of workers in electricity, gas, heat, supply, and water	−0.001	−0.030	0.034
Proportion of workers in information and communications	−0.191	0.175	−0.106
Proportion of workers in transportation and postal services	−0.083	0.080	0.181
Proportion of workers in wholesale and retail trade	−0.126	0.013	−0.136
Proportion of workers in finance and insurance	−0.190	0.126	−0.115
Proportion of workers in real estate and goods rental and leasing	−0.188	0.198	−0.104
Proportion of workers in scientific research, professional and technical services	−0.177	0.143	−0.070
Proportion of workers in accommodations, eating, and drinking services	0.076	0.027	−0.216
Proportion of workers in living-related and personal services and amusement services	0.042	0.038	−0.142
Proportion of workers in education, learning support	−0.024	0.004	−0.100
Proportion of workers in medical, health care and welfare	0.081	−0.025	−0.200
Proportion of workers in compound services	0.232	−0.105	−0.099
Proportion of workers in services, N.E.C.	−0.061	0.146	0.024
Proportion of workers in government, except elsewhere classified	0.188	0.019	−0.036
Proportion of workers in industires unable to classify	−0.151	0.214	−0.060
Proportion of executives of company or corporation	−0.107	0.070	−0.235
Proportion of self-employed workers with employees	0.209	−0.044	−0.133
Proportion of self-employed workers without employees	0.195	−0.115	−0.145
Proportion of family workers	0.215	−0.073	−0.044
Proportion of unknown	−0.131	0.221	−0.086
Proportion of regular employees	0.015	−0.094	0.075
Proportion of dispatched workers from temporary labour agency	−0.169	0.009	0.197
Proportion of part-time workers, contract workers	0.061	0.092	−0.166
Proportion of unemployed persons	0.072	0.064	0.044
Proportion of commuters working in their own cities or villages	0.226	−0.105	−0.038
Proportion of commuters working in other cities or villages within the same prefecture	−0.184	0.050	0.032
Proportion of commuters working in other prefectures	−0.140	0.078	0.051
Proportion of persons aged 60 years and older in population of 15 years and older	0.189	−0.158	−0.115
Proportion of nuclear family household	−0.086	−0.010	0.202
Proportion of extended family household	0.069	−0.198	0.036
Proportion of non-family shared household	−0.080	0.194	0.005
Proportion of one-person household	0.002	0.210	−0.201
Number of restaurants per km^2^	−0.127	0.174	−0.236
Number of large retail stores per km^2^	−0.156	0.183	−0.196
Number of department stores per km^2^	−0.175	0.181	−0.142
Population density per km^2^	−0.157	0.180	−0.159
Number of hospitals per 100,000 persons	0.159	−0.048	−0.082
Number of general clinics per 100,000 persons	−0.044	−0.023	−0.312
Number of dental clinics per 100,000 persons	−0.073	0.131	−0.243
Number of long term care establishments per 100,000 persons aged 65 years and older	0.102	−0.129	0.023
Urbanization promotion areas per km^2^ of habitable areas	−0.192	0.173	−0.077
Urbanization control areas per km^2^ of habitable areas	−0.163	0.037	0.160
Quasi industrial areas per km^2^ of habitable areas	−0.116	0.120	−0.085
Industrial areas per km^2^ of habitable areas	−0.164	0.088	0.056
Exclusively industrial areas per km^2^ of habitable areas	−0.087	0.071	0.150
Commercial areas per km^2^ of habitable areas	−0.144	0.161	−0.215
Proportion of persons with primary/junior high school diploma	0.250	−0.107	0.002
Proportion of persons with high school diploma	0.236	−0.157	0.057
Proportion of persons with some college diploma	−0.230	0.068	0.039
Proportion of persons with undergraduate/Graduate school degree	−0.249	0.123	−0.055
Proportion of persons who walk to work/school per commuter	0.151	0.104	−0.145
Proportion of persons who use their private car to work/school per commuter	0.166	−0.202	0.107
Tax revenue from tabocco per person aged 20 years and older (1,000 yen)	0.091	0.126	−0.005
Taxable income per taxpayer (1,000 yen)	−0.191	0.160	−0.098

**Table 3:** Factor loadings for women’s model.

	LV1	LV2	LV3
Proportion of persons with smoking habit	0.030	0.257	−0.073
Proportion of persons with no physical activity for more than one hour a day	0.009	−0.100	0.032
Proportion of persons with no exercise for more than two weeks	0.119	−0.081	−0.041
Proportion of persons who have dinner within two hours before bedtime more than 3 times a week	−0.058	0.200	0.102
Proportion of persons who drink alcohol everyday	−0.109	0.131	−0.221
Proportion of persons who drink more than 3 glasses (540 ml) of alcohol per day	−0.100	0.239	−0.043
Proportion of persons who drink about 2–3 glasses (360–540 ml) of alcohol per day	−0.062	0.283	−0.087
Proportion of persons who drink about 1–2 glasses (180–360 ml) of alcohol per day	0.017	0.236	−0.081
Proportion of persons who drink less than 1 glass (180 ml) of alcohol per day	0.015	−0.274	0.088
Proportion of persons who snack or drink sweet beverages between meals everyday	−0.071	−0.152	−0.022
Proportion of persons who sometimes snack or drink sweet beverages between meals	0.056	0.072	0.075
Proportion of persons who skip breakfast more than 3 times a week	−0.016	0.298	−0.016
Proportion of persons who are unable to sleep well and enough	−0.109	−0.031	0.099
Proportion of persons who had over 10 kg in body weight gain relative to their body weight in their 20s	0.095	0.214	0.222
Proportion of persons who walk at a faster pace than others of the same age	−0.122	0.142	−0.073
Proportion of persons who are unable to chew food due to problems of tooth, gum, or occlusion	−0.026	0.094	0.101
Proportion of persons with with difficulty chewing food due to problems of tooth, gum, or occlusion	0.140	0.076	−0.007
Proportion of persons who eat quicker than others	0.010	0.006	−0.104
Proportion of persons who eat slower than others	−0.025	−0.019	−0.057
Proportion of workers in agriculture and forestry	0.161	−0.005	−0.052
Proportion of workers in fisheries	0.089	0.087	−0.064
Proportion of workers in mining and quarrying	0.101	−0.008	−0.090
Proportion of workers in construction	0.042	0.005	0.068
Proportion of workers in manufacturing	0.012	−0.140	0.225
Proportion of workers in electricity, gas, heat, supply, and water	−0.041	−0.011	−0.058
Proportion of workers in information and communications	−0.181	0.116	−0.143
Proportion of workers in transportation and postal services	−0.124	0.021	0.224
Proportion of workers in wholesale and retail trade	−0.116	−0.014	0.104
Proportion of workers in finance and insurance	−0.189	0.053	−0.009
Proportion of workers in real estate and goods rental and leasing	−0.199	0.123	−0.114
Proportion of workers in scientific research, professional and technical services	−0.183	0.075	−0.078
Proportion of workers in accommodations, eating, and drinking services	0.063	0.053	−0.238
Proportion of workers in living-related and personal services and amusement services	0.022	0.036	0.064
Proportion of workers in education, learning support	−0.125	−0.075	0.020
Proportion of workers in medical, health care and welfare	0.088	−0.076	−0.059
Proportion of workers in compound services	0.175	−0.092	−0.144
Proportion of workers in services, N.E.C.	−0.143	0.171	−0.027
Proportion of workers in government, except elsewhere classified	0.060	0.032	−0.094
Proportion of workers in industires unable to classify	−0.182	0.152	−0.027
Proportion of executives of company or corporation	−0.048	0.024	−0.264
Proportion of self-employed workers with employees	0.118	0.080	−0.237
Proportion of self-employed workers without employees	0.110	−0.025	−0.246
Proportion of family workers	0.178	−0.028	−0.107
Proportion of unknown	−0.163	0.177	−0.072
Proportion of regular employees	0.082	−0.034	−0.101
Proportion of dispatched workers from temporary labour agency	−0.196	0.061	0.129
Proportion of part-time workers, contract workers	−0.030	0.018	0.069
Proportion of unemployed persons	−0.054	0.178	0.169
Proportion of commuters working in their own cities or villages	0.177	−0.039	−0.097
Proportion of commuters working in other cities or villages within the same prefecture	−0.139	0.013	0.080
Proportion of commuters working in other prefectures	−0.115	0.015	0.110
Proportion of persons aged 60 years and older in population of 15 years and older	0.181	−0.089	−0.172
Proportion of nuclear family household	−0.048	−0.078	0.232
Proportion of extended family household	0.113	−0.142	0.077
Proportion of non-family shared household	−0.110	0.200	0.025
Proportion of one-person household	−0.075	0.205	−0.270
Number of restaurants per km^2^	−0.153	0.134	−0.217
Number of large retail stores per km^2^	−0.174	0.128	−0.161
Number of department stores per km^2^	−0.185	0.114	−0.089
Population density per km^2^	−0.173	0.122	−0.108
Number of hospitals per 100,000 persons	0.124	0.006	−0.101
Number of general clinics per 100,000 persons	−0.038	−0.067	−0.313
Number of dental clinics per 100,000 persons	−0.102	0.100	−0.304
Number of long term care establishments per 100,000 persons aged 65 years and older	0.116	−0.077	0.047
Urbanization promotion areas per km^2^ of habitable areas	−0.192	0.096	−0.030
Urbanization control areas per km^2^ of habitable areas	−0.120	−0.040	0.177
Quasi industrial areas per km^2^ of habitable areas	−0.120	0.090	−0.041
Industrial areas per km^2^ of habitable areas	−0.137	0.032	0.088
Exclusively industrial areas per km^2^ of habitable areas	−0.075	0.046	0.125
Commercial areas per km^2^ of habitable areas	−0.159	0.116	−0.195
Proportion of persons with primary/junior high school diploma	0.204	−0.006	−0.033
Proportion of persons with high school diploma	0.216	−0.051	0.022
Proportion of persons with some college diploma	−0.178	−0.045	0.065
Proportion of persons with undergraduate/Graduate school degree	−0.216	−0.001	−0.023
Proportion of persons who walk to work/school per commuter	0.063	0.145	−0.300
Proportion of persons who use their private car to work/school per commuter	0.188	−0.113	0.093
Tax revenue from tobacco per person aged 20 years and older (1,000 yen)	0.026	0.194	−0.115
Taxable income per taxpayer (1,000 yen)	−0.188	0.073	−0.130

For women, the LV1 (Table 3) was positively loaded by the proportion of persons with a lower level of educational attainment (junior high school or high school), persons commuting with a personal vehicle, family workers, and residents working in their home municipality, while it was negatively loaded by the proportion of persons with a higher level of educational attainment (undergraduate or graduate school), workers in the real estate, finance, information and communications, or scientific research sectors, the number of shops and restaurants, population, and taxable income. The LV2 in women was positively loaded by lifestyle factors such as drinking, smoking, and skipping breakfast and socioenvironmental factors such as the proportion of single-person households, non-relative households, tobacco tax, unemployment, and population density. The negatively loaded socioenvironmental factors for LV3 were the proportion of persons who commute on foot, board members, business owners, the number of general clinics, dental clinics, or restaurants, and commercial areas, while positively loading socioenvironmental factors were the proportion of nuclear family households, workers in manufacturing or transportation sectors, urban control areas, and exclusively industrial areas.

The scatter plots of LV1 and LV2 scores with the proportion of overweight persons in the area present positive relationships, while the scores for LV3 appeared to be clustered (S3 and S4 Figs). The LV1 and LV2 scores and the proportion of overweight individuals in the 335 SMAs were also plotted across 11 regions of Japan, with the color of Region 1 representing the northernmost area and Region 11 representing the southernmost area (S5 and S6 Figs). While many areas in Okinawa exhibited multiple characteristics represented by LV1 and LV2, other areas had higher scores in a specific latent variable.

## Discussion

Our PLS regression analysis identified the LVs contributing to the proportion of overweight persons in SMAs: lower education and income levels and a higher proportion of family workers, a higher proportion of the population living alone combined with unfavorable eating and drinking habits, and a less walkable environment. These LVs had a positive relationship with the proportion of overweight persons in an SMA. Overall, the factor loadings for LV1, LV2, and LV3 were similar between men and women, although factors relating to the regional economic sector profile appeared to be more useful in men than women. Interestingly, among all factors included in our analysis, lifestyle factors were not the most influential factors in most LVs compared to socioenvironmental factors, except for LV2 in women. One possible explanation is that self-reported lifestyle behaviors, such as diet and exercise, were subject to social desirability bias, with respondents potentially overreporting healthy behaviors during health checkups. Additionally, individuals may not be fully aware of their habitual physical activity or other unconscious lifestyle patterns, which limits the accuracy of self-assessments. In contrast, socioenvironmental factors may more reliably reflect these unconscious behaviors, indirectly capturing lifestyle influences that participants themselves may not recognize. These considerations may help explain the relatively lower contribution of self-reported lifestyle variables in LVs.

We will first discuss LV1. The LV1 for both sexes seemed to represent the area where persons with lower educational attainment and lower income live. The LV1 in men included economic sectors of agriculture and construction, while factors related to limited access to food services were included for women. What LV1 represents agrees with prior research at local [28,29] and global [30] levels, though we could not identify similar evidence from Japan.

Some lifestyle factors, such as drinking alcohol and skipping breakfast more than three times a week, and single-person households were common contributions to LV2 in both men and women. Regarding dietary practices, according to the 2019 Japanese National Health and Nutrition Survey, more than 45% of men and women in their 40s responded that they were too busy with work or housework to have healthy eating habits [10]. Alongside unhealthy eating and drinking behaviors, LV2 in the present study was positively loaded by proportion of workers in the information and communications sector and the real estate sector. This suggests that the regions characterized by LV2 tend to have a higher concentration of these industry sectors.

Additionally, other lifestyle factors, such as smoking and having dinner within two hours of going to bed, were also the main contributors to the women’s LV2. For the men’s LV2, better access to stores and restaurants appeared to be important positively-loading factors. Based on the contributors we identified, LV2 may represent a somewhat urban area with a higher population density and a higher proportion of people living alone with unfavorable eating and drinking habits. According to a study in France, the association between neighborhood deprivation and being overweight is stronger in inner-city areas and suburbs of large cities than in rural areas [31]. In our study, LV2 did not show negative loading from the taxable income of the area. Instead, it showed positive loading from the information and communications sector and the real estate sector, which may not align with the well-known description of deprivation and overweight in urban and suburban area. Our study identified unique characteristics of overweight in populated areas compared to findings from previous research.

For men and women, LV3 may represent a less walkable environment considering the negative loading of factors such as commuters on foot, commercial areas, the number of stores, restaurants, clinics, and dentists, combined with workers in the manufacturing and transportation sectors. Urban control areas and exclusively industrial areas were positive loading factors. Walkability is often measured based on residential density, mixed land use, and street connectivity [32]. A study from the U.S. reported the association between the characteristics of a more walkable environment (e.g., more mixed land use, greater street connectivity, and a higher density of residences or recreational facilities) and active transportation [33]. Another study focusing on Toyama prefecture in Japan reported that women residents in walkable neighborhoods had lower BMIs [34]. Our LV3 at the SMA level aligns with these previous results. According to a review, there are associations between the availability of full-service establishments (e.g., supermarkets and non-fast-food restaurants) and metabolic health and obesity. Weak evidence linking proximity to the workplace with metabolic health, and a potential impact of commuting through food environments on metabolic health and weight status were also reported [11]. The characteristics of the area represented by LV3, with negative loadings from the proportion of foot commuters and the density of shops or restaurants, may reflect an inactive daily lifestyle and limited availability of full-service establishments imposed by the built environment. Although our study could not incorporate detailed data on food availability in the environments through which persons commute or the precise distance or duration of travel, factors such as the proportion of individuals working in their residential city and car commuters were positively loading factors in LV1 for both men and women.

In LV1, LV2, and LV3, speed of eating or walking (either slow or fast) and self-reported lack of physical activities or exercise had minimal loading effects in comparison to other factors, which was surprising considering the well-recognized role that physical activity plays in preventing and managing overweight and obesity [35]. This may underscore the importance of unconscious lifestyle choices potentially determined by socioenvironmental factors [13].

Although we cannot claim that the identified LVs are the direct cause of overweight in an SMA, our findings provide valuable insights for planning health promotion interventions considering the characteristics of vulnerable groups in the population. For instance, if one intervention is selected to implement initially, it may be beneficial to prioritize strategies addressing LV1. In preparing such interventions, investigating the lifestyles of those with lower income, lower education levels, and those working in family businesses could help identify specific needs for appropriate weight management programs. Conversely, focusing on enhancing walkability may be more beneficial for areas with low LV1 and LV2 scores.

The limitations are discussed below. First, the factors we identified that contribute to each LV may not necessarily be the cause of overweight. Therefore, those planning targeted interventions are advised to make decisions by incorporating additional evidence. However, our study informs which factors are worth exploring to develop interventions that promote healthy weight maintenance and prevention of lifestyle diseases. Second, the LVs were constructed using open-source data, which may have omitted important factors. Also, some variables were drawn from different years. Nonetheless, the models’ coefficient of determination (R^2^) values were 0.86 for men and 0.94 for women. Third, a limitation concerning the generalizability of the study findings arises from the fact that participants in the special health checkups program may have higher health literacy. For a more comprehensive understanding, the officially published screening rates, stratified by sex, age, and type of insurance, are available online [36,37].

## Conclusion

Our study revealed the previously unexplored regional socioenvironmental factors, including economic sectors, and lifestyle factors associated with overweight in Japan. We identified three latent variables: areas with lower education and income levels, characterized by agricultural and construction sectors; areas showing patterns of more individuals living alone and exhibiting unfavorable eating and drinking habits, linked with information and communications as well as real estate sectors; and areas with less walkable environments, highlighted by the manufacturing industry and transportation sectors. Although this study focused specifically on Japan, it highlights the potential importance of the link between area-level variation in overweight and economic sector profiles. Given the limited international research on this topic, it provides useful insights for broader investigations into the structural determinants of overweight. Addtionally, some areas reflected traits persistent across latent variables, whereas others were more pronounced in a particular latent variable. Policymakers must recognize that there are distinct types of socioenvironmental areas, each with its own potential challenges, when developing interventions to promote healthy lifestyles that manage weight and prevent lifestyle diseases.

## Supporting information

S1 File S1 Table: Details of lifestyle and socioenvironmental variables, denominators used in calculations, and data sources. S2 Table: Descriptive statistics for men’s lifestyle behavioral and socioenvironmental variables at SMA-level (n = 335) S3 Table: Descriptive statistics for women’s lifestyle behavioral and socioenvironmental variables at SMA-level (n = 335).
(DOCX)

S1 Fig Cross-validation of the men’s model using the one-sigma heuristic. (TIF)

S2 Fig Cross-validation of the women’s model using the one-sigma heuristic. (TIF)

S3 Fig Scatter plots of the LV scores and the proportion of men with overweight (BMI ≥ 25 kg/m^2^) in each SMA. (TIF)

S4 Fig Scatter plots of the LV scores and the proportion of women with overweight (BMI ≥ 25 kg/m^2^) in each SMA. (TIF)

S5 Fig LV1 and LV2 scores and the proportion of men with overweight (BMI ≥ 25 kg/m^2^) across 11 regional classifications. (TIF)

S6 Fig LV1 and LV2 scores and the proportion of women with overweight (BMI ≥ 25 kg/m^2^) across 11 regional classifications. (TIF)
