# Supplementary material for: Regional variation in overweight and associations with regional profiles using a Japanese national open-source database
Source: PLoS One. 2025 Aug 25;20(8):e0328435. doi: 10.1371/journal.pone.0328435 (PMC12377602; doi:10.1371/journal.pone.0328435)
Supplement: S1 File — S1 Table: Details of lifestyle and socioenvironmental variables, denominators used in calculations, and data sources. S2 Table: Descriptive statistics for men’s lifestyle behavioral and socioenvironmental variables at SMA-level (n = 335) S3 Table: Descriptive statistics for women’s lifestyle behavioral and socioenvironmental variables at SMA-level (n = 335). (DOCX) [file pone.0328435.s001.docx]

| S1 Table. Details of lifestyle and socioenvironmental variables, denominators used in calculations, and data sources. | | | | | | |
| --- | --- | --- | --- | --- | --- | --- |
| Lifestyle variables | | | | Data source | |  |
| Proportion of persons with specific lifestyle behavior (%) | | Number of persons with smoking habit | | Open-access version of specific health checkups data (2018) | |  |
|  |  | Number of persons with no physical activity for more than one hour a day | |  |  |  |
|  |  | Number of persons with no exercise for more than two weeks | |  |  |  |
|  |  | Number of persons who have dinner within two hours before bedtime more than 3 times a week | |  |  |  |
|  |  | Number of persons who drink alcohol everyday | |  |  |  |
|  |  | Number of persons who drink more than 3 glasses (540 ml) of alcohol per day | |  |  |  |
|  |  | Number of persons who drink about 2-3 glasses (360 – 540 ml) of alcohol per day | |  |  |  |
|  |  | Number of persons who drink about 1-2 glasses (180 – 360 ml) of alcohol per day | |  |  |  |
|  |  | Number of persons who drink less than 1 glass (180 ml) of alcohol per day | |  |  |  |
|  |  | Number of persons who snak or drink sweet beverages between meals everyday | |  |  |  |
|  |  | Number of persons who sometimes snack or drink sweet beverages between meals | |  |  |  |
|  |  | Number of persons who skip breakfast more than 3 times a week | |  |  |  |
|  |  | Number of persons who are unable to sleep well and enough | |  |  |  |
|  |  | Number of persons who had over 10 kg in body weight gain relative to their body weight in their 20s | |  |  |  |
|  |  | Number of persons who walk at a faster pace than others of the same age | |  |  |  |
|  |  | Number of persons who are unable to chew food due to problems of tooth, gum, or occlusion | |  |  |  |
|  |  | Number of persons with difficulty chewing food due to problems of tooth, gum, or occlusion | |  |  |  |
|  |  | Number of persons who eat quicker than others | |  |  |  |
|  |  | Number of persons who eat slower than others | |  |  |  |
|  |  | Denominator: Number of persons who completed health checkups | |  |  |  |
| Socioenvironmental variables | | | | Data source | |  |
| Proportion of workers in each industrial classification (%) | | Number of workers in agriculture and forestry | | Population cencus of Japan (2015) | |  |
|  |  | Number of workers in fisheries | |  |  |  |
|  |  | Number of workers in mining and quarrying | |  |  |  |
|  |  | Number of workers in construction | |  |  |  |
|  |  | Number of workers in manufacturing | |  |  |  |
|  |  | Number of workers in electricity, gas, heat, supply, and water | |  |  |  |
|  |  | Number of workers in information and communications | |  |  |  |
|  |  | Number of workers in transportation and postal services | |  |  |  |
|  |  | Number of workers in wholesale and retail trade | |  |  |  |
|  |  | Number of workers in finance and insurance | |  |  |  |
|  |  | Number of workers in real estate and goods rental and leasing | |  |  |  |
|  |  | Number of workers in scientific research, professional and technical services | |  |  |  |
|  |  | Number of workers in accommodations, eating, and drinking services | |  |  |  |
|  |  | Number of workers in living-related and personal services and amusement services | |  |  |  |
|  |  | Number of workers in education, learning support | |  |  |  |
|  |  | Number of workers in medical, health care and welfare | |  |  |  |
|  |  | Number of workers in compound services | |  |  |  |
|  |  | Number of workers in services, N.E.C. | |  |  |  |
|  |  | Number of workers in government, except elsewhere classified | |  |  |  |
|  |  | Number of workers in industires unable to classify | |  |  |  |
|  |  | Denominator: Total workers between the age of 40 and 74 | |  |  |  |
| Proportion of each employment status (%) | | Number of executives of company or corporation | | Population cencus of Japan (2015) | |  |
|  |  | Number of self-employed workers with employees | |  |  |  |
|  |  | Number of self-employed workers without employees | |  |  |  |
|  |  | Number of family workers | |  |  |  |
|  |  | Number of regular employees | |  |  |  |
|  |  | Number of dispatched workers from temporary labour agency | |  |  |  |
|  |  | Number of part-time workers, contract workers | |  |  |  |
|  |  | Number of unemployed persons | |  |  |  |
|  |  | Number of employment status unknown | |  |  |  |
|  |  | Denominator: Working population | |  |  |  |
| Proportion of workers inside or outside their local city or prefecture (%) | | Number of persons working in their own cities or villages | | Population cencus of Japan (2015) | |  |
|  |  | Number of persons working in other cities or villages within the same prefecture | |  |  |  |
|  |  | Number of persons working in other prefectures | |  |  |  |
|  |  | Denominator: Working population | |  |  |  |
| Proportion of each commuting method (%) | | Number of persons who walk to work/school per commuter | | Population cencus of Japan (2010) | |  |
|  |  | Number of personswho use their private car to work/school per commuter | |  |  |  |
|  |  | Denominator: All commuters | |  |  |  |
| Proportion of each educational level (%) | | Number of persons with primary/junior high school diploma | | Population cencus of Japan (2010) | |  |
|  |  | Number of persons with high school diploma | |  |  |  |
|  |  | Number of persons with some college diploma | |  |  |  |
|  |  | Number of persons with undergraduate/graduate dgree | |  |  |  |
|  |  | Denominator: Total graduates | |  |  |  |
| Taxable income per taxpayer (1,000 yen) | | Taxable income (1,000yen) | | Municipal tax survey* (2018) | |  |
|  |  | Denominator: Number of taxpayer | |  |  |  |
| Tax revenue from tabacco tax per persons aged 20 and older (1,000 yen) | | Tax revenue from tabocco per person aged 20 and older (1,000yen) | | Municipal tax collection survey* (2015) | |  |
|  |  | Denominator: Persons aged 20 years and older | | Population cencus of Japan (2010) | |  |
| Proportion of each household type (%) | | Nuclear family household | | Population cencus of Japan (2015) | |  |
|  |  | Extended family household | |  |  |  |
|  |  | Non-family shared household | |  |  |  |
|  |  | One-person household | |  |  |  |
|  |  | Denominator: All household | |  |  |  |
| Population aged 60 and above (%) | | Persons aged 60 years or older | | Basic Resident Registration (2018) | |  |
|  |  | Denominator: Persons aged 15 years and older | |  |  |  |
| Access to each shop per ㎢ | | Number of restaurants | | Economic census (2016) | |  |
|  |  | Number of large retail stores | |  |  |  |
|  |  | Number of department stores | |  |  |  |
|  |  | Denominator: Habitable areas (㎢) | | Population cencus of Japan, Municipalities Area Statistics of Japan (2015) | |  |
| Access to each health service per 100,000 persons | | Number of hospitals | | Survey of medical institutions , hospital reports (2015) | |  |
|  |  | Number of general clinics | |  |  |  |
|  |  | Number of dental clinics | |  |  |  |
|  |  | Denominator: Total population | | Population cencus of Japan (2015) | |  |
|  |  | Number of long term care establishments | | Survey of institutions and establishments for long-term care (2015) | |  |
|  |  | Denominator: Persons aged 65 years and older | | Population cencus of Japan (2015) | |  |
| Population density (persons per ㎢) | | Population | | Population cencus of Japan (2015) | |  |
|  |  | Denominator: Habitable areas (㎢) | | Population cencus of Japan, Municipalities Area Statistics of Japan (2015) | |  |
| Proportion of areas for city planning (%) | | Urbanization promotion areas (㎢) | | Urban planning annual report* | |  |
|  |  | Urbanization control areas (㎢) | |  |  |  |
|  |  | Quasi industrial areas (㎢) | |  |  |  |
|  |  | Industrial areas (㎢) | |  |  |  |
|  |  | Exclusively industrial areas (㎢) | |  |  |  |
|  |  | Commercial areas (㎢) | |  |  |  |
|  |  | Denominator: Habitable areas (㎢) | | Population cencus of Japan, Municipalities Area Statistics of Japan (2015) | |  |
| * The survey name was tranlated to English by authors. | | | |  | |  |
|  | | | |  | |  |
| S2 Table. Descriptive statistics for men’s lifestyle behavioral and socioenvironmental variables at SMA-level (n = 335) | | | | | | |
|  | Variables | | Mean (SD) | | Median [25%,75%] | |
| Lifestyle variables | | |  | |  | |
|  | Proportion of persons with smoking habit | | 0.34 (0.03) | | 0.34 [0.32, 0.36] | |
|  | Proportion of persons with no physical activity for more than one hour a day | | 0.59 (0.05) | | 0.60 [0.57, 0.62] | |
|  | Proportion of persons with no exercise for more than two weeks | | 0.71 (0.03) | | 0.71 [0.69, 0.73] | |
|  | Proportion of persons who have dinner within two hours before bedtime more than 3 times a week | | 0.33 (0.04) | | 0.33 [0.30, 0.36] | |
|  | Proportion of persons who drink alcohol everyday | | 0.42 (0.05) | | 0.41 [0.38, 0.44] | |
|  | Proportion of persons who drink more than 3 glasses (540 ml) of alcohol per day | | 0.06 (0.02) | | 0.05 [0.04, 0.06] | |
|  | Proportion of persons who drink about 2-3 glasses (360 – 540 ml) of alcohol per day | | 0.18 (0.03) | | 0.17 [0.16, 0.19] | |
|  | Proportion of persons who drink about 1-2 glasses (180 – 360 ml) of alcohol per day | | 0.36 (0.03) | | 0.36 [0.34, 0.39] | |
|  | Proportion of persons who drink less than 1 glass (180 ml) of alcohol per day | | 0.40 (0.06) | | 0.41 [0.38, 0.44] | |
|  | Proportion of persons who snak or drink sweet beverages between meals everyday | | 0.13 (0.02) | | 0.13 [0.12, 0.14] | |
|  | Proportion of persons who sometimes snack or drink sweet beverages between meals | | 0.55 (0.03) | | 0.56 [0.54, 0.57] | |
|  | Proportion of persons who skip breakfast more than 3 times a week | | 0.19 (0.03) | | 0.18 [0.16, 0.21] | |
|  | Proportion of persons who are unable to sleep well and enough | | 0.32 (0.04) | | 0.32 [0.29, 0.35] | |
|  | Proportion of persons who had over 10 kg in body weight gain relative to their body weight in their 20s | | 0.46 (0.03) | | 0.46 [0.44, 0.47] | |
|  | Proportion of persons who walk at a faster pace than others of the same age | | 0.47 (0.04) | | 0.47 [0.45, 0.50] | |
|  | Proportion of persons who are unable to chew food due to problems of tooth, gum, or occlusion | | 0.01 (0.01) | | 0.01 [0.01, 0.01] | |
|  | Proportion of persons with with difficulty chewing food due to problems of tooth, gum, or occlusion | | 0.20 (0.03) | | 0.20 [0.18, 0.22] | |
|  | Proportion of persons who eat quicker than others | | 0.35 (0.02) | | 0.35 [0.34, 0.37] | |
|  | Proportion of persons who eat slower than others | | 0.07 (0.02) | | 0.07 [0.06, 0.07] | |
| Socioenvironmental variables | | |  | |  | |
|  | Proportion of workers in agriculture and forestry | | 0.08 (0.06) | | 0.06 [0.03, 0.11] | |
|  | Proportion of workers in fisheries | | 0.01 (0.03) | | 0.00 [0.00, 0.01] | |
|  | Proportion of workers in mining and quarrying | | 0.00 (0.00) | | 0.00 [0.00, 0.00] | |
|  | Proportion of workers in construction | | 0.14 (0.04) | | 0.13 [0.11, 0.16] | |
|  | Proportion of workers in manufacturing | | 0.19 (0.08) | | 0.18 [0.13, 0.24] | |
|  | Proportion of workers in electricity, gas, heat, supply, and water | | 0.01 (0.00) | | 0.01 [0.01, 0.01] | |
|  | Proportion of workers in information and communications | | 0.02 (0.02) | | 0.01 [0.01, 0.02] | |
|  | Proportion of workers in transportation and postal services | | 0.08 (0.02) | | 0.08 [0.07, 0.09] | |
|  | Proportion of workers in wholesale and retail trade | | 0.12 (0.02) | | 0.12 [0.10, 0.13] | |
|  | Proportion of workers in finance and insurance | | 0.02 (0.01) | | 0.01 [0.01, 0.02] | |
|  | Proportion of workers in real estate and goods rental and leasing | | 0.02 (0.01) | | 0.01 [0.01, 0.02] | |
|  | Proportion of workers in scientific research, professional and technical services | | 0.03 (0.01) | | 0.03 [0.02, 0.04] | |
|  | Proportion of workers in accommodations, eating, and drinking services | | 0.03 (0.02) | | 0.03 [0.02, 0.03] | |
|  | Proportion of workers in living-related and personal services and amusement services | | 0.02 (0.00) | | 0.02 [0.02, 0.02] | |
|  | Proportion of workers in education, learning support | | 0.04 (0.01) | | 0.04 [0.03, 0.04] | |
|  | Proportion of workers in medical, health care and welfare | | 0.05 (0.01) | | 0.05 [0.04, 0.06] | |
|  | Proportion of workers in compound services | | 0.01 (0.01) | | 0.01 [0.01, 0.02] | |
|  | Proportion of workers in services, N.E.C. | | 0.07 (0.01) | | 0.07 [0.06, 0.08] | |
|  | Proportion of workers in government, except elsewhere classified | | 0.05 (0.02) | | 0.05 [0.04, 0.06] | |
|  | Proportion of workers in industires unable to classify | | 0.03 (0.02) | | 0.02 [0.01, 0.04] | |
|  | Proportion of executives of company or corporation | | 0.06 (0.01) | | 0.06 [0.05, 0.07] | |
|  | Proportion of self-employed workers with employees | | 0.03 (0.01) | | 0.03 [0.03, 0.04] | |
|  | Proportion of self-employed workers without employees | | 0.12 (0.05) | | 0.11 [0.08, 0.15] | |
|  | Proportion of family workers | | 0.02 (0.01) | | 0.02 [0.01, 0.03] | |
|  | Proportion of employment status unknown | | 0.03 (0.02) | | 0.02 [0.01, 0.03] | |
|  | Proportion of regular employees | | 0.82 (0.02) | | 0.82 [0.81, 0.83] | |
|  | Proportion of dispatched workers from temporary labour agency | | 0.02 (0.01) | | 0.02 [0.02, 0.03] | |
|  | Proportion of part-time workers, contract workers | | 0.16 (0.02) | | 0.15 [0.14, 0.17] | |
|  | Proportion of unemployed persons | | 0.05 (0.01) | | 0.05 [0.04, 0.05] | |
|  | Proportion of commuters working in their own cities or villages | | 0.62 (0.19) | | 0.65 [0.49, 0.77] | |
|  | Proportion of commuters working in other cities or villages within the same prefecture | | 0.29 (0.15) | | 0.27 [0.18, 0.40] | |
|  | Proportion of commuters working in other prefectures | | 0.07 (0.10) | | 0.03 [0.01, 0.07] | |
|  | Proportion of persons aged 60 years and older in population of 15 years and older | | 0.31 (0.05) | | 0.31 [0.28, 0.34] | |
|  | Proportion of nuclear family household | | 0.56 (0.05) | | 0.57 [0.54, 0.59] | |
|  | Proportion of extended family household | | 0.12 (0.06) | | 0.11 [0.07, 0.16] | |
|  | Proportion of non-family shared household | | 0.01 (0.00) | | 0.01 [0.01, 0.01] | |
|  | Proportion of one-person household | | 0.31 (0.06) | | 0.30 [0.26, 0.34] | |
|  | Number of restaurants per ㎢ | | 7.88 (16.76) | | 3.05 [1.75, 6.02] | |
|  | Number of large retail stores per ㎢ | | 0.23 (0.43) | | 0.09 [0.03, 0.19] | |
|  | Number of department stores per ㎢ | | 0.02 (0.03) | | 0.01 [0.00, 0.02] | |
|  | Population density per ㎢ | | 1682.28 (2914.34) | | 690.84 [374.92, 1376.95] | |
|  | Number of hospitals per 100,000 persons | | 8.39 (4.10) | | 7.40 [5.40, 10.57] | |
|  | Number of general clinics per 100,000 persons | | 77.55 (18.94) | | 75.35 [65.32, 87.35] | |
|  | Number of dental clinicsper 100,000 persons | | 48.52 (12.67) | | 47.57 [42.04, 52.39] | |
|  | Number of long term care establishments per 100,000 persons aged 65 years and older | | 13.81 (5.62) | | 13.52 [9.97, 16.81] | |
|  | Urbanization promotion areas per ㎢ of habitable areas | | 0.17 (0.25) | | 0.07 [0.00, 0.23] | |
|  | Urbanization control areas per ㎢ of habitable areas | | 0.33 (0.41) | | 0.15 [0.00, 0.59] | |
|  | Quasi industrial areas per ㎢ of habitable areas | | 0.02 (0.05) | | 0.01 [0.00, 0.02] | |
|  | Industrial areas per ㎢ of habitable areas | | 0.01 (0.01) | | 0.01 [0.00, 0.01] | |
|  | Exclusively industrial areas per ㎢ of habitable areas | | 0.01 (0.02) | | 0.00 [0.00, 0.02] | |
|  | Commercial areas per ㎢ of habitable areas | | 0.02 (0.03) | | 0.01 [0.00, 0.02] | |
|  | Proportion of persons with primary/junior high school diploma | | 0.22 (0.09) | | 0.22 [0.16, 0.28] | |
|  | Proportion of persons with high school diploma | | 0.67 (0.13) | | 0.70 [0.60, 0.78] | |
|  | Proportion of persons with some college diploma | | 0.12 (0.02) | | 0.12 [0.10, 0.13] | |
|  | Proportion of persons with undergraduate/Graduate school degree | | 0.13 (0.06) | | 0.12 [0.09, 0.16] | |
|  | Proportion of persons who walk to work/school per commuter | | 0.07 (0.03) | | 0.06 [0.05, 0.08] | |
|  | Proportion of persons who use their private car to work/school per commuter | | 0.61 (0.19) | | 0.68 [0.57, 0.74] | |
|  | Tax revenue from tabocco per person aged 20 years and older (1,000 yen) | | 9.02 (1.71) | | 8.75 [8.07, 9.64] | |
|  | Taxable income per taxpayer (1,000 yen) | | 2982.66 (503.79) | | 2887.69 [2653.68, 3165.52] | |
|  |  | |  | |  | |

| S3 Table. Descriptive statistics for women’s lifestyle behavioral and socioenvironmental variables at SMA-level (n = 335) | | | |
| --- | --- | --- | --- |
|  | Variables | Mean (SD) | Median [25%,75%] |
| Lifestyle variables | |  |  |
|  | Proportion of persons with smoking habit | 0.09 (0.03) | 0.09 [0.08, 0.11] |
|  | Proportion of persons with no physical activity for more than one hour a day | 0.58 (0.07) | 0.58 [0.54, 0.62] |
|  | Proportion of persons with no exercise for more than two weeks | 0.76 (0.03) | 0.76 [0.73, 0.78] |
|  | Proportion of persons who have dinner within two hours before bedtime more than 3 times a week | 0.18 (0.03) | 0.18 [0.16, 0.19] |
|  | Proportion of persons who drink alcohol everyday | 0.12 (0.02) | 0.12 [0.11, 0.14] |
|  | Proportion of persons who drink more than 3 glasses (540 ml) of alcohol per day | 0.01 (0.01) | 0.01 [0.00, 0.01] |
|  | Proportion of persons who drink about 2-3 glasses (360 – 540 ml) of alcohol per day | 0.04 (0.02) | 0.04 [0.03, 0.05] |
|  | Proportion of persons who drink about 1-2 glasses (180 – 360 ml) of alcohol per day | 0.18 (0.04) | 0.17 [0.15, 0.20] |
|  | Proportion of persons who drink less than 1 glass (180 ml) of alcohol per day | 0.77 (0.06) | 0.78 [0.74, 0.81] |
|  | Proportion of persons who snak or drink sweet beverages between meals everyday | 0.29 (0.04) | 0.29 [0.26, 0.33] |
|  | Proportion of persons who sometimes snack or drink sweet beverages between meals | 0.57 (0.04) | 0.57 [0.55, 0.60] |
|  | Proportion of persons who skip breakfast more than 3 times a week | 0.11 (0.03) | 0.11 [0.09, 0.12] |
|  | Proportion of persons who are unable to sleep well and enough | 0.36 (0.04) | 0.36 [0.34, 0.38] |
|  | Proportion of persons who had over 10 kg in body weight gain relative to their body weight in their 20s | 0.28 (0.03) | 0.28 [0.26, 0.30] |
|  | Proportion of persons who walk at a faster pace than others of the same age | 0.44 (0.04) | 0.44 [0.41, 0.46] |
|  | Proportion of persons who are unable to chew food due to problems of tooth, gum, or occlusion | 0.00 (0.00) | 0.00 [0.00, 0.00] |
|  | Proportion of persons with with difficulty chewing food due to problems of tooth, gum, or occlusion | 0.16 (0.02) | 0.16 [0.14, 0.18] |
|  | Proportion of persons who eat quicker than others | 0.28 (0.02) | 0.28 [0.26, 0.29] |
|  | Proportion of persons who eat slower than others | 0.09 (0.02) | 0.09 [0.08, 0.09] |
| Socioenvironmental variables | |  |  |
|  | Proportion of workers in agriculture and forestry | 0.07 (0.06) | 0.05 [0.02, 0.10] |
|  | Proportion of workers in fisheries | 0.00 (0.01) | 0.00 [0.00, 0.00] |
|  | Proportion of workers in mining and quarrying | 0.00 (0.00) | 0.00 [0.00, 0.00] |
|  | Proportion of workers in construction | 0.03 (0.01) | 0.03 [0.03, 0.03] |
|  | Proportion of workers in manufacturing | 0.13 (0.05) | 0.12 [0.09, 0.16] |
|  | Proportion of workers in electricity, gas, heat, supply, and water | 0.00 (0.00) | 0.00 [0.00, 0.00] |
|  | Proportion of workers in information and communications | 0.01 (0.01) | 0.00 [0.00, 0.01] |
|  | Proportion of workers in transportation and postal services | 0.02 (0.01) | 0.02 [0.01, 0.02] |
|  | Proportion of workers in wholesale and retail trade | 0.17 (0.02) | 0.17 [0.16, 0.19] |
|  | Proportion of workers in finance and insurance | 0.02 (0.01) | 0.02 [0.02, 0.03] |
|  | Proportion of workers in real estate and goods rental and leasing | 0.01 (0.01) | 0.01 [0.01, 0.02] |
|  | Proportion of workers in scientific research, professional and technical services | 0.02 (0.01) | 0.02 [0.01, 0.02] |
|  | Proportion of workers in accommodations, eating, and drinking services | 0.08 (0.02) | 0.08 [0.07, 0.09] |
|  | Proportion of workers in living-related and personal services and amusement services | 0.05 (0.01) | 0.05 [0.04, 0.05] |
|  | Proportion of workers in education, learning support | 0.06 (0.01) | 0.05 [0.05, 0.06] |
|  | Proportion of workers in medical, health care and welfare | 0.22 (0.04) | 0.21 [0.19, 0.24] |
|  | Proportion of workers in compound services | 0.01 (0.00) | 0.01 [0.01, 0.01] |
|  | Proportion of workers in services, N.E.C. | 0.05 (0.01) | 0.05 [0.04, 0.06] |
|  | Proportion of workers in government, except elsewhere classified | 0.02 (0.01) | 0.02 [0.02, 0.02] |
|  | Proportion of workers in industires unable to classify | 0.03 (0.02) | 0.02 [0.01, 0.04] |
|  | Proportion of executives of company or corporation | 0.03 (0.01) | 0.03 [0.02, 0.03] |
|  | Proportion of self-employed workers with employees | 0.01 (0.00) | 0.01 [0.01, 0.01] |
|  | Proportion of self-employed workers without employees | 0.05 (0.01) | 0.04 [0.04, 0.05] |
|  | Proportion of family workers | 0.09 (0.04) | 0.08 [0.05, 0.12] |
|  | Proportion of employment status unknown | 0.02 (0.02) | 0.02 [0.01, 0.03] |
|  | Proportion of regular employees | 0.47 (0.05) | 0.46 [0.43, 0.51] |
|  | Proportion of dispatched workers from temporary labour agency | 0.03 (0.01) | 0.03 [0.02, 0.04] |
|  | Proportion of part-time workers, contract workers | 0.50 (0.05) | 0.51 [0.47, 0.54] |
|  | Proportion of unemployed persons | 0.03 (0.01) | 0.03 [0.03, 0.04] |
|  | Proportion of commuters working in their own cities or villages | 0.71 (0.16) | 0.74 [0.61, 0.84] |
|  | Proportion of commuters working in other cities or villages within the same prefecture | 0.23 (0.13) | 0.22 [0.13, 0.33] |
|  | Proportion of commuters working in other prefectures | 0.03 (0.06) | 0.01 [0.00, 0.04] |
|  | Proportion of persons aged 60 years and older in population of 15 years and older | 0.38 (0.06) | 0.38 [0.34, 0.42] |
|  | Proportion of nuclear family household | 0.56 (0.05) | 0.57 [0.54, 0.59] |
|  | Proportion of extended family household | 0.12 (0.06) | 0.11 [0.07, 0.16] |
|  | Proportion of non-family shared household | 0.01 (0.00) | 0.01 [0.01, 0.01] |
|  | Proportion of one-person household | 0.31 (0.06) | 0.30 [0.26, 0.34] |
|  | Number of restaurants per ㎢ | 7.88 (16.76) | 3.05 [1.75, 6.02] |
|  | Number of large retail stores per ㎢ | 0.23 (0.43) | 0.09 [0.03, 0.19] |
|  | Number of department stores per ㎢ | 0.02 (0.03) | 0.01 [0.00, 0.02] |
|  | Population density per ㎢ | 1682.28 (2914.34) | 690.84 [374.92, 1376.95] |
|  | Number of hospitals per 100,000 persons | 8.39 (4.10) | 7.40 [5.40, 10.57] |
|  | Number of general clinics per 100,000 persons | 77.55 (18.94) | 75.35 [65.32, 87.35] |
|  | Number of dental clinicsper 100,000 persons | 48.52 (12.67) | 47.57 [42.04, 52.39] |
|  | Number of long term care establishments per 100,000 persons aged 65 years and older | 13.81 (5.62) | 13.52 [9.97, 16.81] |
|  | Urbanization promotion areas per ㎢ of habitable areas | 0.17 (0.25) | 0.07 [0.00, 0.23] |
|  | Urbanization control areas per ㎢ of habitable areas | 0.33 (0.41) | 0.15 [0.00, 0.59] |
|  | Quasi industrial areas per ㎢ of habitable areas | 0.02 (0.05) | 0.01 [0.00, 0.02] |
|  | Industrial areas per ㎢ of habitable areas | 0.01 (0.01) | 0.01 [0.00, 0.01] |
|  | Exclusively industrial areas per ㎢ of habitable areas | 0.01 (0.02) | 0.00 [0.00, 0.02] |
|  | Commercial areas per ㎢ of habitable areas | 0.02 (0.03) | 0.01 [0.00, 0.02] |
|  | Proportion of persons with primary/junior high school diploma | 0.22 (0.09) | 0.22 [0.16, 0.28] |
|  | Proportion of persons with high school diploma | 0.67 (0.13) | 0.70 [0.60, 0.78] |
|  | Proportion of persons with some college diploma | 0.12 (0.02) | 0.12 [0.10, 0.13] |
|  | Proportion of persons with undergraduate/Graduate school degree | 0.13 (0.06) | 0.12 [0.09, 0.16] |
|  | Proportion of persons who walk to work/school per commuter | 0.07 (0.03) | 0.06 [0.05, 0.08] |
|  | Proportion of persons who use their private car to work/school per commuter | 0.61 (0.19) | 0.68 [0.57, 0.74] |
|  | Tax revenue from tabocco per person aged 20 years and older (1,000 yen) | 9.02 (1.71) | 8.75 [8.07, 9.64] |
|  | Taxable income per taxpayer (1,000 yen) | 2982.66 (503.79) | 2887.69 [2653.68, 3165.52] |
|  |  |  |  |
