# Supplementary figures and images for: Regional variation in overweight and associations with regional profiles using a Japanese national open-source database
Source: PLoS One. 2025 Aug 25;20(8):e0328435. doi: 10.1371/journal.pone.0328435 (PMC12377602; doi:10.1371/journal.pone.0328435)

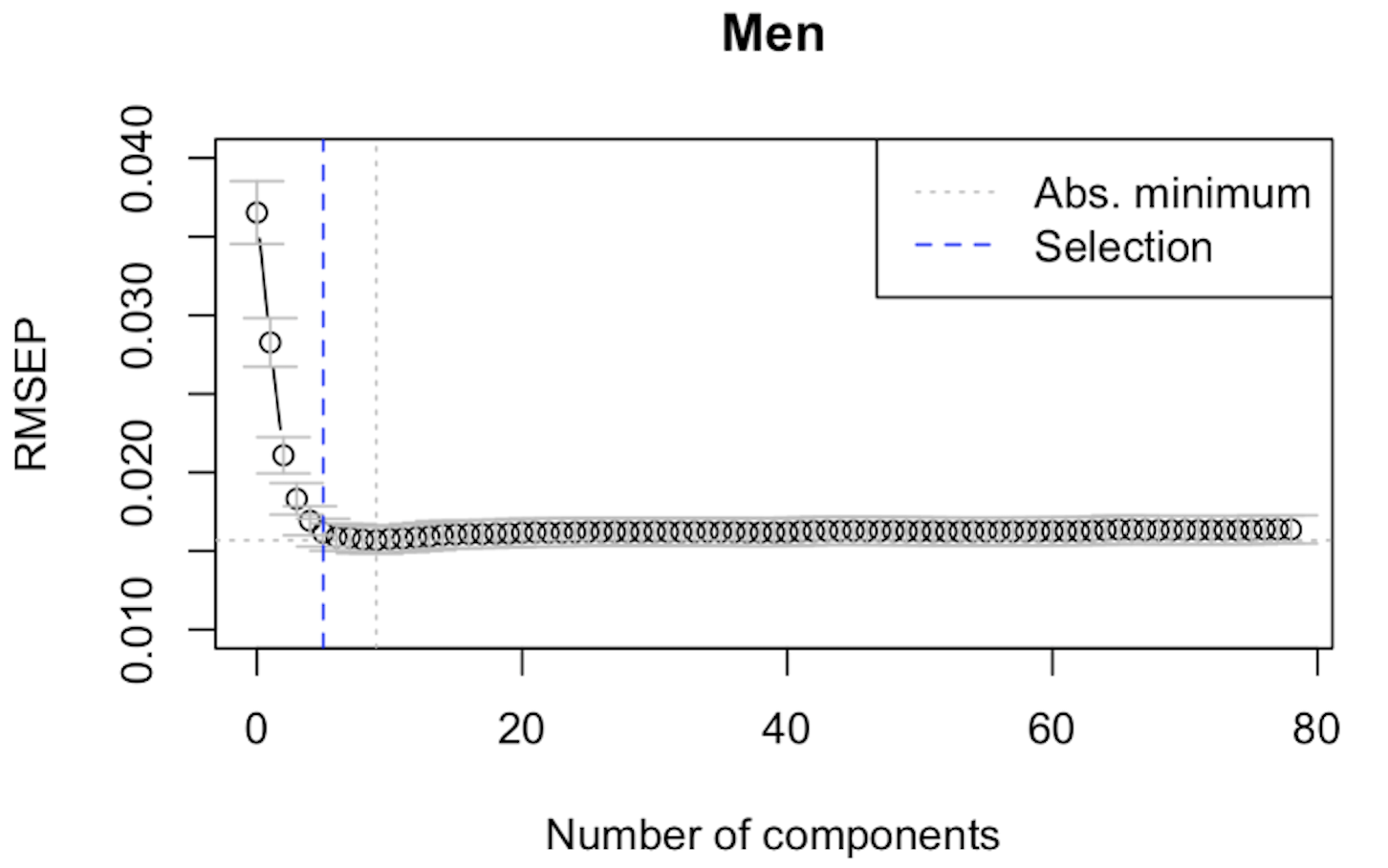

Supplement: S1 Fig — (TIF) [file pone.0328435.s002.tif]

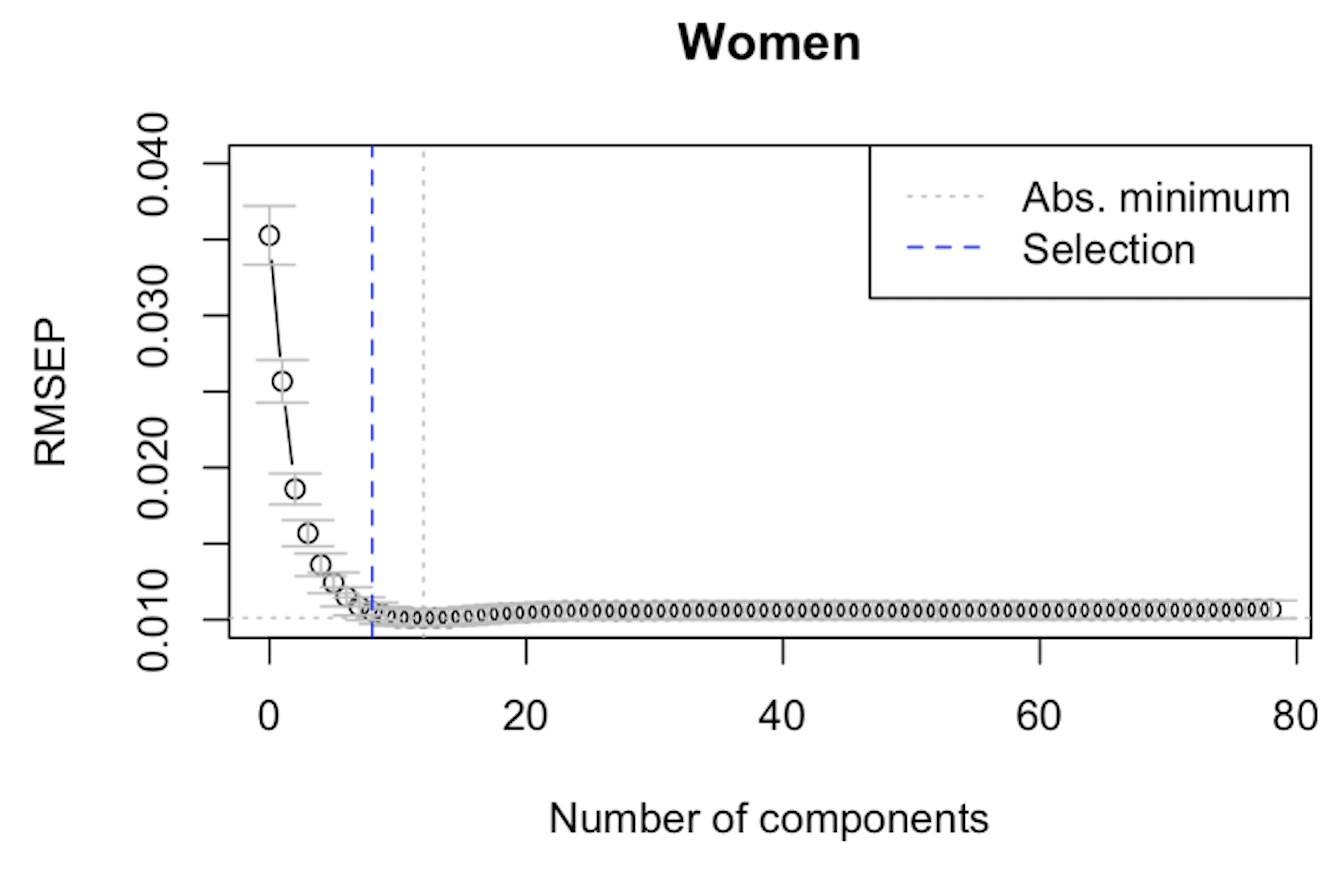

Supplement: S2 Fig — (TIF) [file pone.0328435.s003.tif]

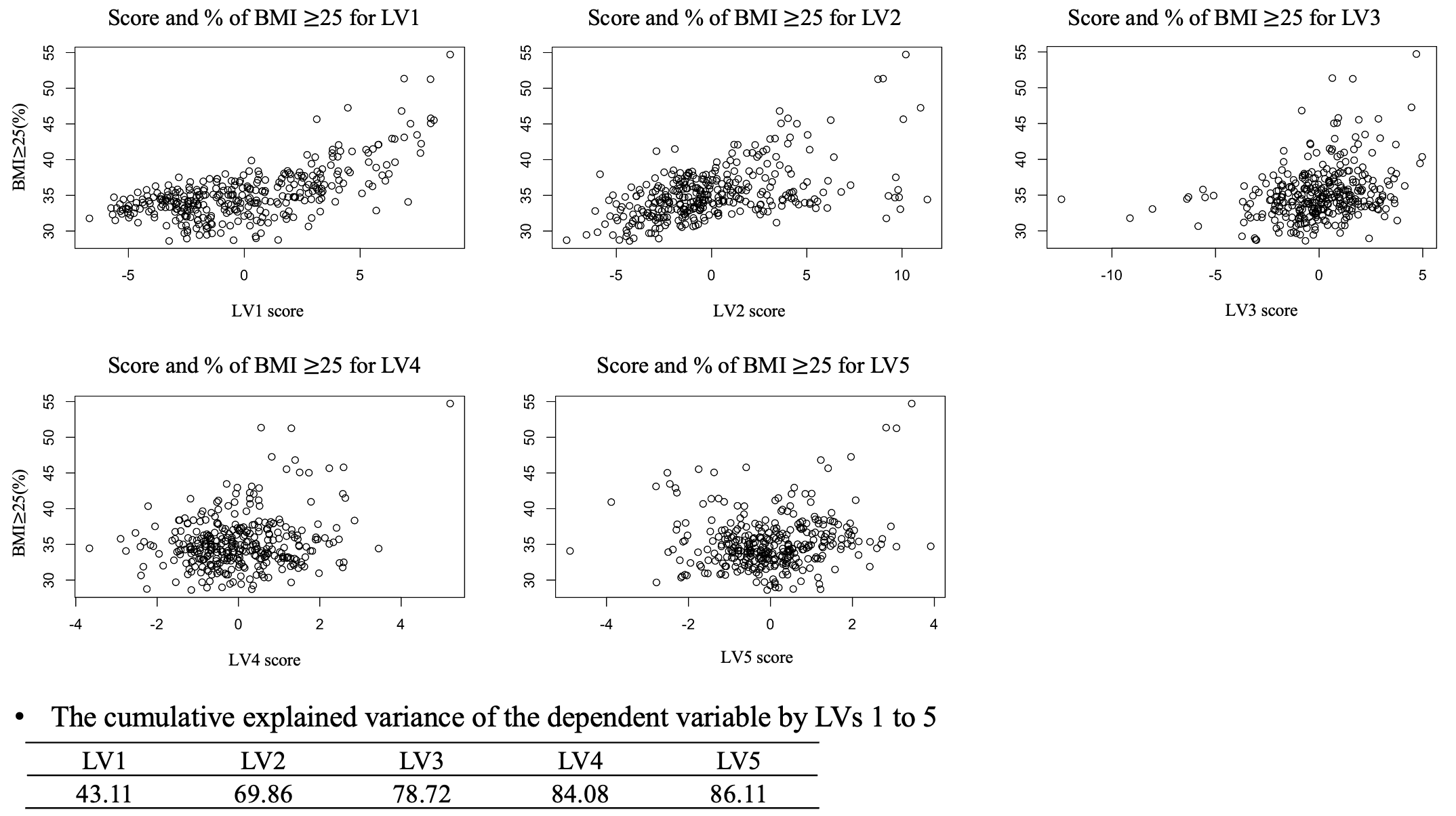

Supplement: S3 Fig — (TIF) [file pone.0328435.s004.tif]

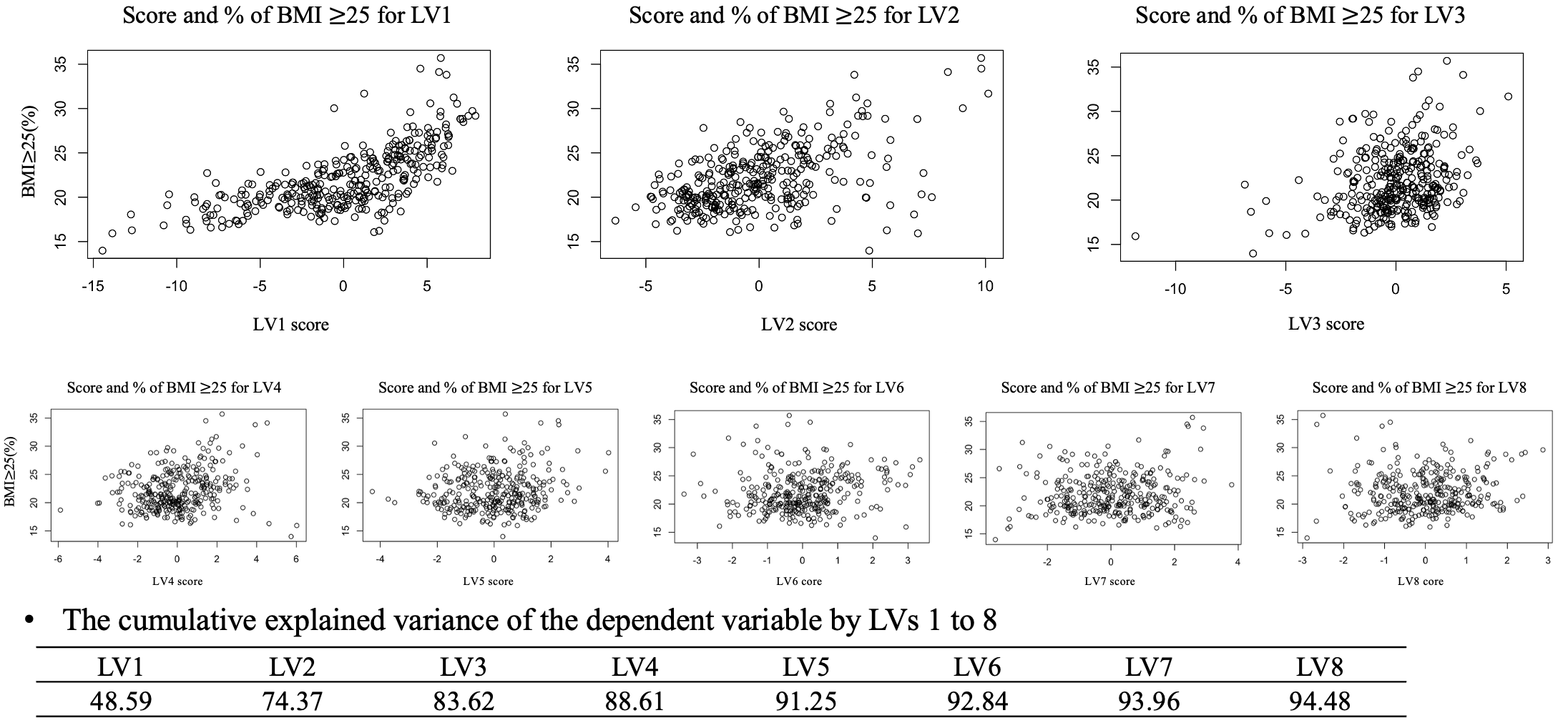

Supplement: S4 Fig — (TIF) [file pone.0328435.s005.tif]

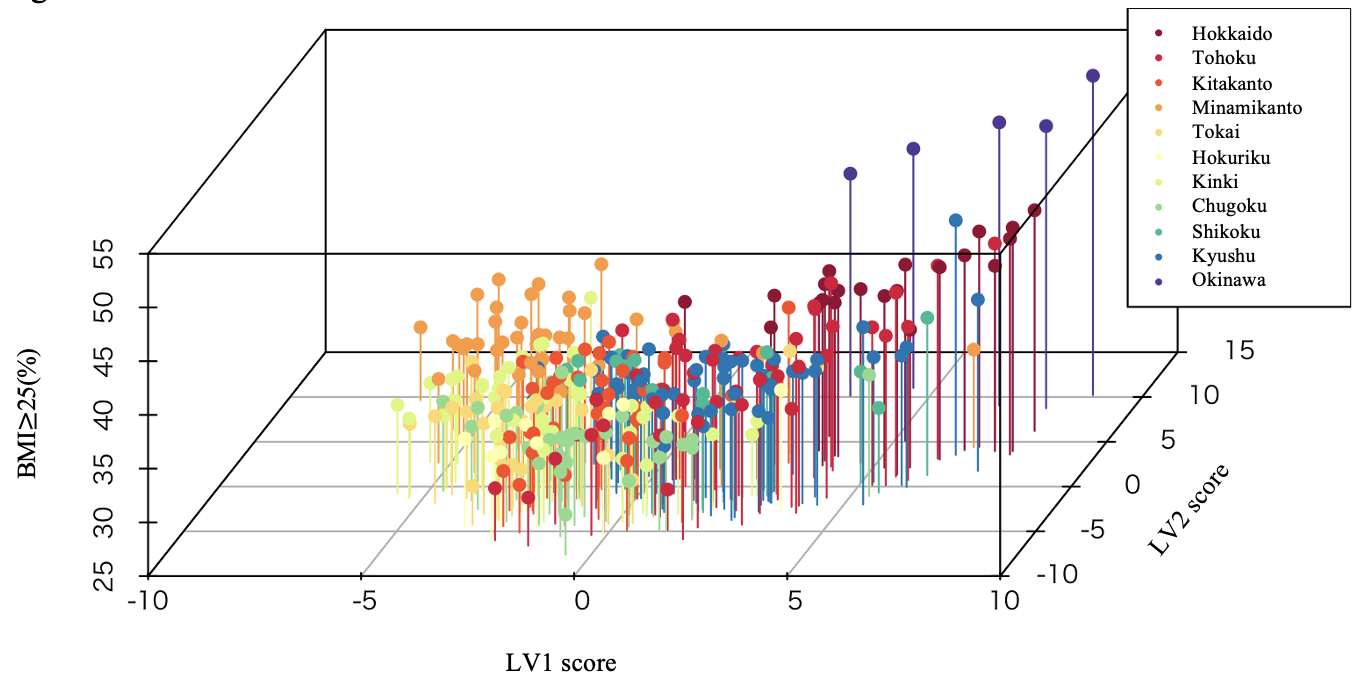

Supplement: S5 Fig — (TIF) [file pone.0328435.s006.tif]

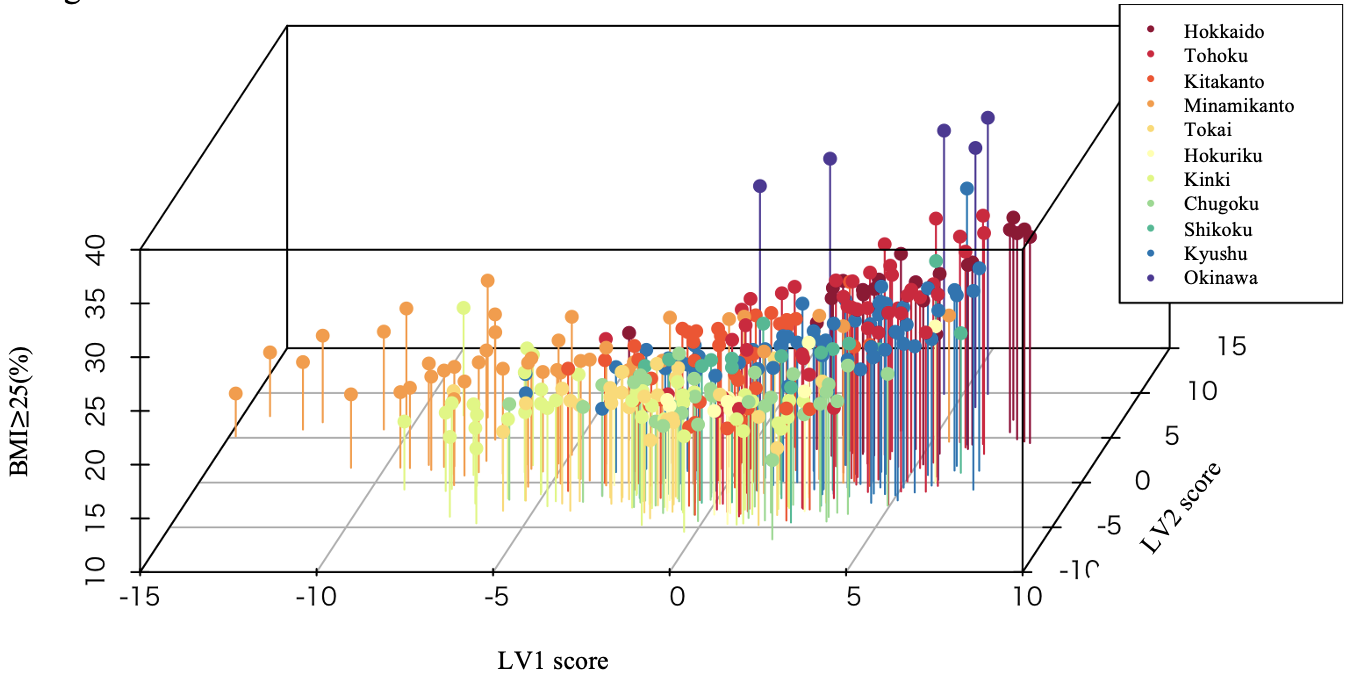

Supplement: S6 Fig — (TIF) [file pone.0328435.s007.tif]
